# Pattern of recurrence in patients with a pathologically complete response after neoadjuvant chemoradiotherapy and surgery for oesophageal cancer

**DOI:** 10.1093/bjsopen/zrab022

**Published:** 2021-04-20

**Authors:** M de Jongh, B M Eyck, L R van der Werf, E L A Toxopeus, J J B van Lanschot, S M Lagarde, A van der Gaast, J Nuyttens, B P L Wijnhoven

**Affiliations:** Department of Surgery, Erasmus MC University Medical Centre, Rotterdam, the Netherlands; Department of Surgery, Erasmus MC University Medical Centre, Rotterdam, the Netherlands; Department of Surgery, Erasmus MC University Medical Centre, Rotterdam, the Netherlands; Department of Surgery, Erasmus MC University Medical Centre, Rotterdam, the Netherlands; Department of Surgery, Erasmus MC University Medical Centre, Rotterdam, the Netherlands; Department of Surgery, Erasmus MC University Medical Centre, Rotterdam, the Netherlands; Department of Medical Oncology, Erasmus MC University Medical Centre, Rotterdam, the Netherlands; Department of Radiation Oncology, Erasmus MC Cancer Institute, Rotterdam, the Netherlands; Department of Surgery, Erasmus MC University Medical Centre, Rotterdam, the Netherlands

## Abstract

**Background:**

Neoadjuvant chemoradiotherapy (nCRT) and surgery is a widely used treatment for locally advanced resectable oesophageal cancer, with 20–50 per cent of patients having a pathological complete response (pCR). Disease, however, still recurs in 20–30 per cent of these patients. The aim of this study was to assess the pattern of recurrence in patients with a pCR after nCRT and surgery.

**Methods:**

All patients with a pCR after nCRT and surgery included in the phase II and III CROSS (ChemoRadiotherapy for Oesophageal followed by Surgery Study) trials (April 2001 to December 2008) and after the CROSS trials (September 2009 to October 2017) were identified. The site of recurrence was compared with the applied radiation and surgical fields. Outcomes were median time to recurrence, and overall and progression-free survival.

**Results:**

A total of 141 patients with a median follow-up of 100 (i.q.r. 64–134) months were included. Some 29 of 141 patients (20,6 per cent) developed recurrence. Of these, four had isolated locoregional recurrence, 15 had distant recurrence only, and ten had both locoregional and distant recurrence. Among the 14 patients with locoregional recurrences, five had recurrence within the radiation field, seven outside the radiation field, and two at the border. Median time to recurrence was 24 (10–62) months. The 5-year overall survival rate was 74 per cent and the recurrence-free survival rate was 70 per cent.

**Conclusion:**

Despite good overall survival, recurrence still occurred in 21 per cent of patients. Most recurrences were distant, outside the radiation and surgical fields.

## Teaser

Although patients with a pathological complete response after neoadjuvant chemoradiotherapy and surgery have a good prognosis, recurrence still develops in 21 per cent. Most recurrences were either distant or located outside the radiation and surgical field, which stresses the need for more effective systemic therapy.

## Introduction

Oesophageal cancer is the eighth most common cancer worldwide, with an incidence rate of 4.7–11.5 per 100 000 person-years^1^. Neoadjuvant chemoradiotherapy (nCRT) followed by surgery is widely used for patients with locally advanced resectable oesophageal cancer. In many countries, the nCRT regimen consists of carboplatin and paclitaxel combined with concurrent 41.4 Gy radiotherapy, based on the CROSS trial^2^^,^^3^. Besides improved survival compared with surgery alone, the CROSS trial showed that 23 per cent of patients with oesophageal adenocarcinoma (OAC) and 49 per cent with squamous cell carcinoma (SCC) had a pathological complete response (pCR) after nCRT, defined by absence of residual vital tumour cells in the surgical resection specimen^2^.

It is hypothesized that nCRT reduces locoregional recurrence by downstaging the primary tumour, facilitating resection with tumour-negative resection margins. Although nCRT may also affect locoregional lymph nodes, there remains a risk of recurrence outside the radiation field. Several studies^4–9^ have shown that 13–29 per cent of patients with a pCR develop locoregional and/or distant recurrences, although the studies are difficult to compare as they involved a variety of chemotherapeutic agents, as well as different radiation doses, fractions, and fields.

Better insight into the location and timing of recurrence in patients with a pCR after nCRT may guide the development of more effective locoregional or systemic treatment options. The aim of this study was to assess the pattern of recurrence in patients with a pCR after nCRT and surgery using the most widely used regimen: CROSS^2^. 

## Methods

This was a cohort study involving all patients with oesophageal cancer who underwent nCRT according to the CROSS regimen followed by surgery at Erasmus MC between September 2009 and October 2017, identified from a prospectively developed institutional database. Information on patients included in the phase II and III CROSS trials between April 2001 and December 2008 at Erasmus MC and at other participating centres was retrieved from the study database^2^^,^^10^. Patients were included in this study if a pCR was reported after pathological examination of the resection specimen. Patients were excluded if they had not completed at least 80 per cent of the nCRT or did not subsequently undergo oesophagectomy.

### Neoadjuvant chemoradiotherapy and surgery

Patients received nCRT according to the CROSS regimen. This consisted of intravenous carboplatin (dose titration to achieve an area under the curve of 2 mg per m per min) and paclitaxel (50 mg per m^2^ of body surface area), both administered on days 1, 8, 15, 22, and 29. Concurrently, a total dose of 41.4 Gy radiotherapy was given in 23 fractions of 1.8 Gy, 5 days per week, starting on the first day of chemotherapy. A three-dimensional conformal radiation technique was used for external beam radiotherapy. The gross tumour volume was defined by the primary tumour and enlarged regional lymph nodes. The planning target volume comprised a 1.5-cm radial margin and 4-cm proximal and distal margins from the primary tumour. The distal margin was 3 cm if the tumour extended into the stomach^2^.

The preferred surgical approach was open, hybrid or total minimally invasive transthoracic oesophagectomy with two-field lymphadenectomy. For reconstruction, a gastric tube was preferred. Transhiatal oesophagectomy was performed only in patients with a junctional tumour or in those with poor performance status, with the intent to limit surgical trauma and reduce the risk of complications.

### Pathological examination

Pathological examination of the resection specimen was carried out according to a standardized protocol^11^. If no tumour was found macroscopically, the area of scar tissue was embedded in paraffin for microscopic examination. Each block was divided into sections 5 µm thick, and stained with haematoxylin and eosin. A pCR was defined by the absence of residual vital tumour cells in the resection specimen, either at the primary tumour site or in any of the resected lymph nodes. Additional immunohistochemistry was used only when there was uncertainty about undetected isolated tumour cells in the resected specimen. All pathology reports from patients treated at Erasmus MC were retrieved from the patient records and checked for accurate response assessment^12^. Patients from other centres were included in the CROSS trial, and resection specimens were examined according to the trial protocol, with particular attention to assessment of response to chemoradiation^10^.

### Follow-up

Follow-up was done according to the Dutch guideline for oesophageal cancer^13^. Patients were seen in the outpatient clinic every 3 months during the first year, every 6 months during the second year, and yearly thereafter until 5 years after surgery. No routine imaging or endoscopy was performed. Recurrence was sought on the basis of clinical suspicion, with additional imaging (CT of the chest–abdomen, full-body PET–CT, MRI, endoscopic or external ultrasound examination) as deemed appropriate. In all patients, cytological or histological proof of recurrence was sought whenever possible. The last day of follow-up was 31 December 2018 for patients included in the CROSS trials, and 31 October 2019 for those treated at Erasmus MC outside the CROSS trials.

### Outcomes

Outcomes were the rate of recurrences in patients with a pCR, the location of recurrences relative to surgical and radiation fields, time to recurrence, and overall and recurrence-free survival rates. Time to recurrence was measured from the date of surgery to the date of first detection of recurrence. Overall survival was calculated from date of surgery until death or date of last follow-up, and recurrence-free survival from the date of surgery until date of first detection of recurrence or date of death from any cause. Recurrence in relation to the surgical field was defined as locoregional recurrence when found in the mediastinum, at the location of the anastomosis, or in the supraclavicular or coeliac lymph nodes. Recurrence at any other site was defined as distant recurrence. Concurrent locoregional and distant recurrence was defined as recurrence detected at imaging at the same time during follow-up. To determine the location of locoregional recurrences relative to the radiation field, the target area for radiotherapy was reassessed and compared with the CT images that showed recurrence by an expert radiation oncologist. These recurrences were defined as within the field, outside the field or on the border of the radiation field.

### Statistical analysis

Patient and treatment characteristics are reported as numbers with percentages for categorical variables, and median (i.q.r.) for continuous variables. Survival was compared between patients with and without recurrence, and between histological subgroups, using Cox proportional hazards models. Cumulative incidence functions were estimated for illustration of the absolute risk of recurrence, adjusted for competing events. For analysis of the cumulative incidence of recurrence at any site, death before developing a recurrence was considered as a competing risk. For analysis of the cumulative incidence of recurrence at a specific site (locoregional or distant), death before developing a recurrence and recurrence at a site other than the site of interest were considered as a competing risk. In all analyses, two-sided *P* < 0.050 was considered statistically significant. Statistical analyses were performed using R version 3.6.1 (R Foundation for Statistical Computing, Vienna, Austria), using the survival and cmprsk packages.

## Results

**Fig. 1:**
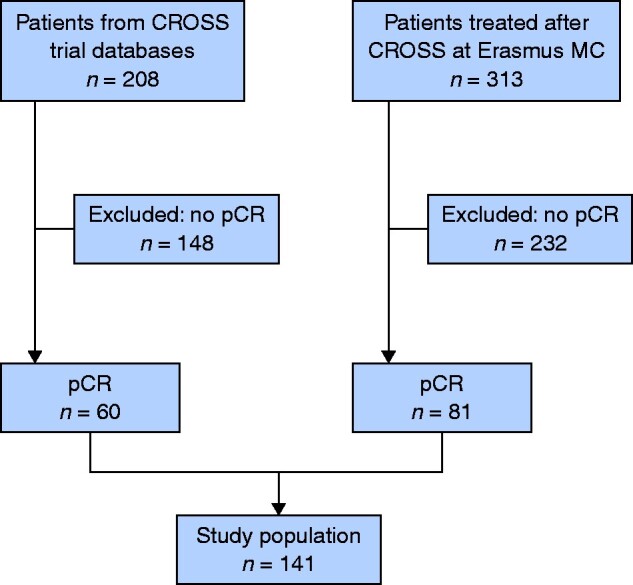
Study flow chart pCR, pathological complete response.

In the phase II and III CROSS trials, 60 of 208 patients (28.8 per cent) had a pCR, whereas 81 of 313 patients (25.8 per cent) in the post-CROSS group had a pCR. Therefore, a total of 141 of 521 screened patients (27.1 per cent) had a pCR and were included in the present study (*Fig. 1*). The median age was 64 (i.q.r. 58–69) years, 98 of 141 patients (69.5 per cent) were men, and 85 (60.2 per cent) had OAC. Patient and treatment characteristics are shown in *Table 1*.

**Table 1:** Patient characteristics

	Total	Adenocarcinoma	Squamous cell carcinoma
	**(*n* = 141)** ^†^	(*n* = 85)	(*n* = 55)
**Age (years)** ^*^	64 (58–69)	63 (57–70)	65 (59–69)
**Sex ratio (M : F)**	98 : 43	70 : 15	28 : 27
**Bodyweight (kg)** ^*^	81 (70–90)	83 (75–95)	72 (61–84)
**Clinical T category**			
cT1	9 (6)	9 (11)	0 (0)
cT2	34 (24)	18 (21)	16 (29)
cT3	96 (68)	57 (67)	38 (69)
cT4	2 (1)	1 (1)	1 (2)
**Clinical N category**			
cN0	54 (38)	37 (44)	17 (31)
cN1	58 (42)	29 (34)	29 (53)
cN2	26 (18)	19 (22)	7 (13)
cN3	1 (1)	0 (0)	1 (2)
Unknown	1 (1)	0 (0)	1 (2)
**Differentiation grade**			
Well	1 (1)	1 (1)	0 (0)
Moderate	58 (41)	32 (38)	26 (47)
Poor	54 (38)	38 (45)	16 (29)
Undifferentiated	1 (1)	1 (1)	0 (0)
Unknown	27 (19)	12 (14)	14 (25)
**Tumour location** ^†^			
Middle oesophagus	35 (25)	6 (7)	29 (53)
Lower oesophagus	83 (59)	57 (67)	25 (45)
Oesophagogastric junction	23 (16)	22 (26)	1 (2)
**Surgical approach**			
Transthoracic oesophagectomy	100 (71)	48 (56)	51 (93)
Transhiatal oesophagectomy	41 (30)	37 (44)	4 (7)
**No. of lymph nodes resected** ^*^	17 (12–23)	15 (11–20)	21 (15–29)

Values in parentheses are percentages unless indicated otherwise;

* values are median (i.q.r.).

† A total of 141 patients, including one with a neuroendocrine tumour.

‡ The mid-thoracic part of the oesophagus is defined as the proximal half of the part between the tracheal bifurcation and the oesophagogastric junction, whereas the lower thoracic portion is the distal half of this part of the oesophagus. Oesophagogastric junctional tumours are lesions whose centre lies within 5 cm proximal or distal to the oesophagogastric junction.

### Pattern of recurrence

Of 141 patients with a pCR, 29 (21 per cent) developed a recurrence, including 21 of 85 patients (25 per cent) with OAC, 7 of 55 (13 per cent) with SCC, and one patient with a neuroendocrine tumour. Some 14 of 141 patients (10 per cent) had a locoregional recurrence, including four with isolated locoregional recurrence and 10 with synchronous distant metastases. Fifteen of 141 patients (11 per cent) had distant metastases only.

Of 41 patients who underwent surgery by a transhiatal approach, 12 (29 per cent) developed recurrence (0 locoregional only, 7 distant only, 5 locoregional and distant). Of the 100 who underwent surgery by a transthoracic approach, 17 developed recurrence (4 locoregional only, 8 distant only, 5 locoregional and distant) (*Table 2*). There was no difference in likelihood of recurrence between the two approaches (odds ratio 2.02, 95 per cent c.i. 0.86 to 4.73; *P* = 0.102).

**Table 2:** Site of recurrence by surgical approach

	Transthoracic oesophagectomy	Transhiatal oesophagectomy
Locoregional only	4	0
Locoregional and distant	5	5
Distant only	8	7
Total	17	12

The distribution of locoregional and distant recurrences relative to the radiation field is shown in *Table 3*. Locoregional recurrences were seen within the radiation field in five of 141 patients (4 per cent), outside the field in seven (5 per cent), and at the border in two patients (1 per cent). Recurrences within the radiation field and at the border of the radiation field were noted only in patients with OAC. All recurrences of SCC developed outside the radiation field.

**Table 3:** Recurrence in relation to radiation field in 141 patients

Recurrence	Within field	Outside field	At border	Total
Locoregional only	1	2	1	4
Locoregional and distant	4	5	1	10
Distant only	0	15	0	15
Total	5	22	2	29

In total, 58 sites of recurrences were recorded in 29 patients. The most common sites of distant recurrence were lungs (11 patients), distant lymph nodes (9), and liver (6). Locoregional recurrences developed mainly in the mediastinum (8 patients), supraclavicular area (4), and coeliac axis (3) (*Table 4*).

**Table 4:** Sites of recurrence

	No. of patients
Locoregional recurrence	
Supraclavicular	4
Coeliac trunk	3
Mediastinum	8
**Distant recurrence**	
Lung	11
Distant lymph node	9
Liver	6
Pleura	4
Bone	4
Cutis/muscle	3
Brain	3
Peritoneum	2
Adrenal gland	1

A total of 58 sites of recurrence were found in 29 patients.

**Fig. 2:**
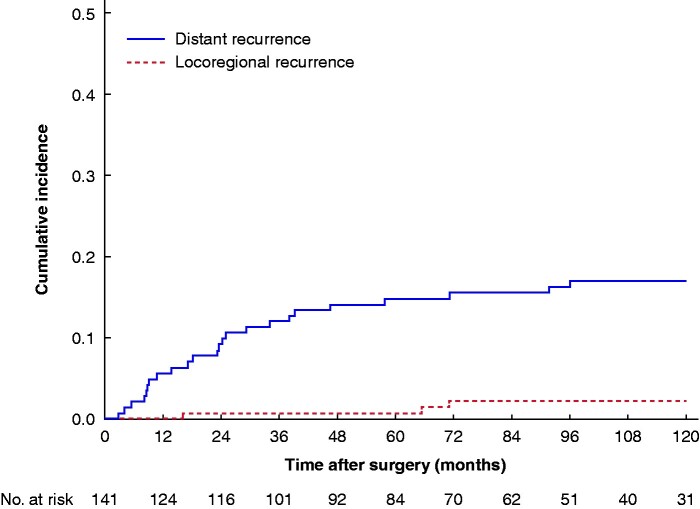
Cumulative incidence of recurrence stratified by location (synchronous distant and locoregional recurrence or distant recurrence alone *versus* locoregional recurrence alone)

The cumulative incidence of recurrence is shown in *Fig. 2*. The median time to recurrence was 24 (i.q.r. 10–62) months, with the majority (22 of 29 patients) being identified within 48 months of surgery, although isolated locoregional recurrences were detected up to 72 months after treatment. The median time to recurrence was 25 (16–71)  months for patients with SCC and 23 (9–52) months for those with OAC.

### Survival

The median follow-up time for surviving patients was 100 (i.q.r. 64–134) months. Among all patients with a pCR, the 5-year overall survival rate was 74 per cent and the recurrence-free survival rate was 70 per cent. At the time of data collection, 6 of 41 patients with recurrence were alive. Patients without recurrence had better overall survival those known to have recurrence (hazard ratio (HR) 0.22, 95 per cent c.i. 0.11 to 0.42; *P* < 0.001), with 5-year overall survival rates of 82 and 39 per cent respectively. Patients with SSC had better overall survival than those with OAC (HR 0.53, 0.29 to 0.98; *P* = 0.041), but there was no difference in overall survival after transhiatal and transthoracic oesophagectomy (HR 0.60, 0.34 to 1.05; *P* = 0.071).

## Discussion

In the present study, 141 of 521 patients (27 per cent) achieved a pCR after nCRT. Despite this, 29 of these patients (21 per cent) developed recurrence at a median of 24 (i.q.r. 10–62) months after surgery. The majority of recurrences were distant and only four patients (3 per cent) developed isolated locoregional recurrence. Recurrences within and at the border of the radiation field were seen exclusively in patients with OAC.

Most patients developed recurrence outside the radiation field, so it seems unlikely that intensifying neoadjuvant radiation or extending the surgical resection would have a large impact in terms of improving survival. In the CROSS trial^3^, patients with SCC responded better to nCRT than those with OAC, resulting in better survival, as confirmed here. The present study showed that, even among patients with a pCR, however, 24 per cent with OAC and 9 per cent with SCC developed distant recurrence. Greater efforts should be made in the development of effective systemic treatments.

Although both histological tumour types were treated with the same neoadjuvant therapy in the present study, the favoured surgical approach for SCC was transthoracic oesophagectomy; 93 per cent of such patients received this approach, compared with 56 per cent of those with OAC. A previous study^14^ indicated that transthoracic oesophagectomy with extended lymphadenectomy only increased survival compared with transhiatal oesophagectomy with limited lymphadenectomy in patients who did not undergo nCRT before surgery. Among patients who had nCRT and surgery, there was no difference in survival between the two surgical approaches.

The greater distant recurrence rate and poorer overall survival of patients with adenocarcinoma in the present study reinforces the need for effective systemic treatment for this histological subtype. Promising results from the FLOT4-AIO (Arbeitsgemeinschaft Internistische Onkologie) trial^15^, with perioperative chemotherapy consisting of fluorouracil plus leucovorin, oxaliplatin and docetaxel, and future results from the Neo-AEGIS (NEOadjuvant trial in Adenocarcinoma of the oEsophagus and oesophagoGastric junction International Study)^16^ and ESOPEC^17^ randomized trials involving OAC may shed more light on this issue. The FLOT regimen may also increase locoregional control for OAC as the pCR rate was remarkably high in patients treated with FLOT (17.4 per cent) and even higher (30 per cent) in patients with tumours at the oesophagogastric junction or with intestinal-type histology^18^.

This study has several limitations. It involved two different databases. The trial database included patients from both inside and outside Erasmus MC. The institutional database comprised 70 per cent of the study patients, and included only patients from Erasmus MC, which may limit the generalizability of the findings. In addition, the number recurrences among patients with a pCR was relatively low, which may have increased the risk of a type II error in the analyses. Not all recurrences were confirmed by histology; some were reported as ‘most likely recurrence’ based on imaging alone, although nearly all of these patients died within the next 17 months. The range of follow-up in the study is wide (24–219 months), which could have led to underestimation of recurrence rates in the more recently included patients. In the present study, no PET–CT was performed after nCRT to exclude distant metastases before surgery. Recent studies have shown that, when such a policy is applied, 8–10 per cent of patients have detectable distant metastases such that surgery is withheld. Recurrence rates after surgery, even in patients with a pCR, could have been lower in the present series if this policy had been applied. Although a limited mediastinal nodal dissection in transhiatal oesophagectomy may have missed tumour-positive lymph nodes in the upper mediastinum, positive nodes in patients with complete pathological regression of the primary tumour are relatively rare, occurring in only 3 per cent of patients^19^, so it seems unlikely that this would have had a significant effect on the results. Survival among patients with recurrence was poor. The majority of these patients do not qualify for treatment with curative intent as most recurrences develop at multiple (distant) sites. Local treatment including radiotherapy may be offered to selected patients with isolated locoregional recurrence, bearing in mind that previous nCRT before surgery often reduces the possibility of reirradiating locoregional recurrence within the previous field.

Response rates and patterns of recurrence differ between the two histological subtypes of oesophageal cancer. Despite this, distant recurrence is most common, suggesting that better systemic rather than locoregional treatments will be key to achieving better survival.

*Disclosure*. The authors declare no conflict of interest. 
